# Identifying residential neighbourhood types from settlement points in a machine learning approach

**DOI:** 10.1016/j.compenvurbsys.2018.01.004

**Published:** 2018-05

**Authors:** Warren C. Jochem, Tomas J. Bird, Andrew J. Tatem

**Affiliations:** WorldPop, Department of Geography and Environment, University of Southampton, UK; Flowminder Foundation, Stockholm, Sweden

**Keywords:** Urban morphology, Land use, Texture, Point pattern analysis, Machine learning, Big data

## Abstract

Remote sensing techniques are now commonly applied to map and monitor urban land uses to measure growth and to assist with development and planning. Recent work in this area has highlighted the use of textures and other spatial features that can be measured in very high spatial resolution imagery. Far less attention has been given to using geospatial vector data (i.e. points, lines, polygons) to map land uses. This paper presents an approach to distinguish residential settlement types (regular vs. irregular) using an existing database of settlement points locating structures. Nine data features describing the density, distance, angles, and spacing of the settlement points are calculated at multiple spatial scales. These data are analysed alone and with five common remote sensing measures on elevation, slope, vegetation, and nighttime lights in a supervised machine learning approach to classify land use areas. The method was tested in seven provinces of Afghanistan (Balkh, Helmand, Herat, Kabul, Kandahar, Kunduz, Nangarhar). Overall accuracy ranged from 78% in Kandahar to 90% in Nangarhar. This research demonstrates the potential to accurately map land uses from even the simplest representation of structures.

## Highlights

• Residential areas can be classified based on spatial patterns detected in a large database of point locations of structures.
• Features summarising density, distance, angles, and spacing are calculated across multiple spatial scales from the points.
• Multi-step variable selection is used to avoid bias in random forest variable importance scores.
• Supervised machine learning approach reaches 78% to 90% classification accuracy.

## 1 Introduction

As populations around the world become more urbanised, particularly in developing countries, the ability to quantify and study the growth and changing function of cities in detail has become more important for urban growth, informal settlements, poverty, environmental and health concerns (Duque, Patino, Ruiz, & Pardo-Pascual, 2015; Herold, Liu, & Clarke, 2003; Kuffer, Pfeffer, & Sliuzas, 2016; Kuffer, Pfeiffer, Sliuzas, & Baud, 2016; UN Habitat, 2016). Moreover, the Sustainable Development Goals (United Nations, 2014) and the New Urban Agenda (United Nations, 2017) have brought additional focus for policymakers on land use planning to create resilient, sustainable, and inclusive cities. To meet such goals, data on intra-urban differences in land uses is needed. Yet the speed of population growth and urbanisation makes it necessary to explore new approaches to assist in producing timely and accurate data on cities and regions.

Recent work to identify land use types across large urban areas has increasingly made use of advances in very high spatial resolution satellite or aerial imagery (Cheriyadat, 2014; Kuffer & Barros, 2011). Similar analyses using large collections of geospatial vector data (points, lines, polygons) have received far less attention in the literature than remote sensing approaches, though several studies have noted the potential to identify classes of buildings or urban land uses (Barr, Barnsley, & Steel, 2004; Hecht, Meinel, & Buchroithner, 2015; Longley & Mesev, 2000; Steiniger, Lange, Burghardt, & Weibel, 2008). Classifying land uses, whether based on imagery or vector data, all rely on the assumption of linking observed spatial forms with different functions or land uses on the ground (Barr et al., 2004). The method developed here uses an existing vector dataset of points representing dwellings (referred to here as settlement points) and applies various measures to quantify the multi-scale, spatial patterns to establish that link and train a machine learning algorithm. The goal is to identify areas of different settlement types and to predict those types into unmapped areas. Land use polygon features (Government of the Islamic Republic of Afghanistan [GoIRA] & UN Habitat, 2015) provide training data for a two-class typology of regular and irregular housing. We then describe several metrics calculated from the spatial point patterns of settlement points which are used to characterise the density and distribution of settlements. While the point geometry-derived features alone provide remarkable accuracy in predicting these classes in our case study of seven provinces in Afghanistan, incorporating additional measures of vegetation, elevation, slope, and nighttime lights improves overall accuracy of the classification, reaching up to 90% accuracy. We identify several spatial relationship measures and scales that were most effective in differentiating residential classes in our study area. Our methodology contributes to ongoing developments in computational methods for utilising big geospatial data, and we implement a variable selection algorithm to select from a large number of correlated features. This work also demonstrates several paths forward for future research. Pattern analysis of vector data can be combined with other remotely sensed data to enhance analyses, as we show with several commonly available satellite-derived measures. Overall the results suggest that our method has potential to extract meaningful information from even the simplest geometric representation of structures.

### 1.1 Remote sensing of urban landscape

To provide timely monitoring of cities and to classify urban land use over large areas, most research has utilised remotely sensed data from airborne or space-borne sensors. Projects such as the Global Human Settlement Layer (Pesaresi et al., 2013) and the Global Urban Footprint (Esch et al., 2013) have expanded this type of monitoring to global scales. Work has also expanded at more local spatial scales, attempting to classify, and monitor land uses within urban areas (Graessar et al., 2012; Kuffer & Barros, 2011) or to extract buildings and identify settled areas (Cheriyadat, 2014; Gros & Tiecke, 2016; Yuan, 2016). These local scale methods have been applied to monitoring informal housing in developing countries by measuring the distinctive patterns of small, dense, irregularly shaped, agglomerative structures (Graessar et al., 2012; Kit, Lüdeke, & Reckien, 2012; Kuffer, Pfeffer, & Sliuzas, 2016; Kuffer, Pfeiffer, Sliuzas, & Baud, 2016). Research in this area of intra-urban classification is notable for its methodological shift away from pixel-based spectral measures (e.g. vegetation indices) toward object-oriented feature extraction and spatial and textural measures that take advantage of patterns and textures detectable in very high spatial resolution (VHR) imagery (Herold et al., 2003; Tatem & Hay, 2004). The most commonly used textures in recent urban mapping applications include entropy, contrast, variance and other measures calculated on the grey level co-occurrence matrix (GLCM; Haralick, Shanmugam, & Dinstein, 1973) and related metrics (Pesaresi & Gerhardinger, 2011; Pesaresi, Gerhardinger, & Kayitakire, 2008) that delineate built-up areas (Duque et al., 2015; Kuffer, Pfeiffer, Sliuzas, & Baud, 2016; Owen & Wong, 2013). Other work has made use of lacunarity measures to quantify the spacing between structures (Kit et al., 2012) as well as the distribution and orientation of line segments extracted from the image (Engstrom et al., 2015). The complexity of urban settlement patterns often requires multiple metrics to be used together and different size filters or feature calculation windows to measure characteristics expressed at different spatial scales (Graessar et al., 2012). The growth in the availability of both VHR imagery and computing power needed to process it has made this area of research very active in recent years.

### 1.2 Vector data analyses of urban areas

Similar to the increasing availability of remote sensing datasets, geospatial vector data (i.e. points, lines, polygons) are now commonly collected and maintained for urban areas by government agencies as part of planning, topographic map production, and tax records, by commercial data providers, or even by volunteers as in the OpenStreetMap project (http://www.openstreetmap.org). These databases have varying degrees of completeness (Hecht, Kunze, & Hahmann, 2013), but, when they can provide comprehensive coverage of urban infrastructure, they offer an alternative approach from remote sensing imagery to monitor urban form and land uses.

In their richest and most complete form, vector databases can construct complete digital, 3D city models containing representations of individual structures. Such a model can add important information on building height to 2D representations on maps (Sridharan & Qiu, 2013). These databases can be time consuming and difficult to construct, however, and research has focused on building them through automated extraction from aerial photographs or LIDAR data (Rottensteiner & Briese, 2002). Other common vector data formats are 2D polygons delineating building footprints as commonly seen on topographic maps and cadastral surveys. While indicating the size and shape of a structure, these data rarely provide other information on land use or building height unless they can be linked with property data or tax records. In their most basic form, buildings can be represented as individual point features. Such settlement points (sometimes called dwelling unit points or address points) are most conventionally used to improve geocoding accuracy (Zandbergen, 2008), but they have also been used as ancillary datasets to identify settled areas for population distribution models (Zandbergen, 2011).

Yet characteristics of vector geometries can be indicative of land use in local areas. This idea requires an alternative interpretation of geospatial vector data – rather than representing discrete objects, the mapped shapes act as markers that, taken together as a pattern, identify broader or more general features of the built landscape. According to Steiniger et al. (2008), spatial pattern recognition of urban land uses adheres to principles of Gestalt psychology and human perceptions of form. When we view a topographic map, for example, we not only see individual structures, we also interpret patterns based on the proximity and similarity among objects to recognize concepts such as “suburbs” or “city centres.” Quantifying these patterns with building density, size, shape, and orientation can enable us to train more realistic, automated classifications (Steiniger et al., 2008). In developing such an interpretation of spatial data, Barr et al. (2004) distinguish between categories of “morphological properties” and “spatial relations” to organise shape measurements. The first category includes geometric attributes such as area (volume in 3D) or compactness of the shape. The latter group of spatial relationships or spatial structures includes measures of proximity or connectivity between vector objects which can be quantified with the number of edges and distances between nodes on a Gabriel graph or other spanning tree structure (Barr et al., 2004). This idea of pattern recognition and classification in spatial data has been taken up particularly by cartographers seeking to identify building types and to automate map generalisations (Hecht et al., 2015; Li, Yan, Ai, & Chen, 2004; Lüscher & Weibel, 2013; Steiniger et al., 2008; Zhang, Ai, Stoter, Kraak, & Molenaar, 2013).

In contrast to the studies discussed above, which all use 2D or 3D polygon representations of buildings, Longley and Mesev (2000) and Mesev, 2005, Mesev, 2007 demonstrated the use of point representations of structures for similar classification goals. Using address points of several UK cities from an Ordnance Survey database and point pattern statistics of density and nearest neighbour index, they identified measureable differences between UK neighbourhood types corresponding with construction years. The types of measures that can be calculated from point data are necessarily limited. Morphological properties such as compactness are not available for point geometries. Only spatial structures can be calculated and even then connectivity of the building structures (e.g. buildings sharing a wall) cannot be observed.

This current study emerges from the research stream of studies such as Mesev, 2005, Mesev, 2007 which use point-level vector data representing structures, yet its objectives are more closely aligned to those of remote sensing-based image classification of settlement areas, such as Graessar et al. (2012). Unlike previous vector data analyses (e.g. Barr et al., 2004; Hecht et al., 2015), the goal here is not to classify building features themselves into types, but to derive a surface classifying areas of particular settlement types. We begin with a land use map which covers portions of major cities in Afghanistan, yet we want to predict those basic categories for residential types in other areas. Section 2 develops a set of measures of spatial interrelationships between points which are calculated across scales and then used as data features in a machine learning method to classify settlement area types. The processing steps are computationally intensive and we discuss several steps to improve efficiency through parallelisation. We demonstrate our methods with data from seven provinces in Afghanistan.

## 2 Methods and data

The goal of this work is to derive a surface classifying inhabited, residential areas into regular or irregular settlement types on a 20 m spatial resolution grid. Regular settlements are characterised by formal planning and regularly spaced and arranged houses and roads as part of large-scale urban planning. Irregular settlements, conversely, have an unplanned layout that includes narrower and irregular growth of roads and houses. Irregular settlements typically lack basic services and often occupy less desirable land along urban fringes and areas vulnerable to hazards. This classification is derived from the available training data (GoIRA & UN Habitat, 2015) as discussed below.

**Fig. 1:**
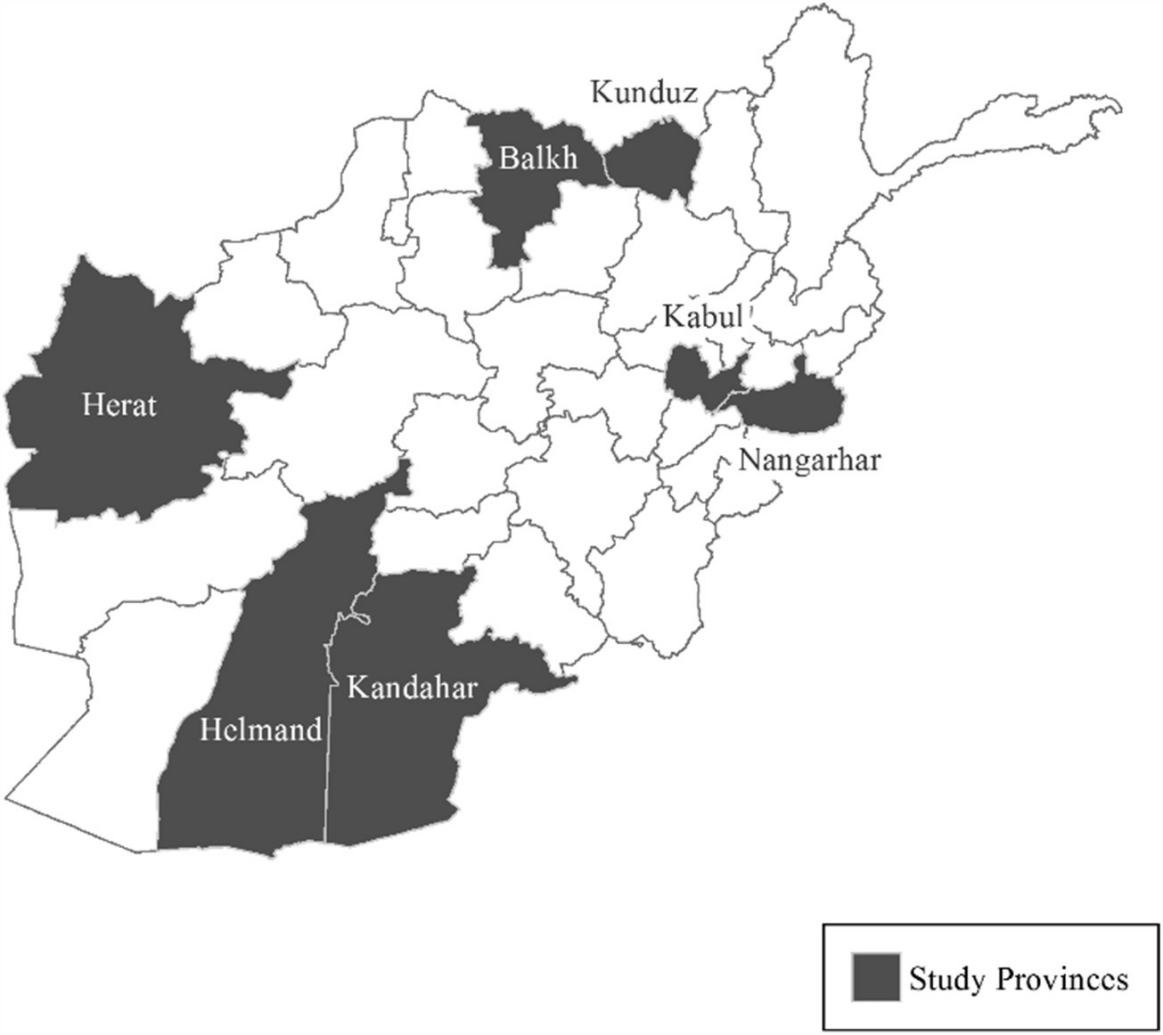
Seven study provinces within Afghanistan. Training locations are selected from within land use maps of the provincial capitals.

The output resolution was selected based on preliminary analyses using the settlement points that found an average distance separating points of approximately 20 m. The supervised classification uses a random forest (RF) approach and relies on multiscale feature indices calculated from the distribution of settlement point locations. The method is tested using the point feature-derived measures alone and in combination with remotely sensed measures in seven provinces (Fig. 1). The provinces were selected: 1) to include major urban areas, 2) for the availability of independent training and validation data, and 3) to include sites from different areas of the country. The RF model is trained using data drawn from all seven areas, and the predictions are evaluated separately for each province.

### 2.1 Training and validation data

In order to perform supervised classification, training locations on regular and irregular residential land uses were drawn from the *State of Afghan Cities* (SoAC) data set (GoIRA & UN Habitat, 2015). The SoAC 2014/2015 programme was initiated by the national government to provide the first study of urbanisation in Afghanistan. This project included extensive collection and mapping of dwelling and land uses in the 34 provincial capitals. Land use was classified into 23 sub-classes of built-up and non-built-up areas, by manually digitizing areas on high resolution imagery (≤61 cm) by trained local analysts. Imagery acquisition dates were 2013–2014 for all areas used in the present study. As part of the SoAC programme, the digitized land use maps were then verified in field visits and in meetings with local community members. Regular and irregular residential settlement classes were created by grouping several land use subtypes, as summarised in Table 1. There are many different terms used in the literature for urban contexts such as “slum” or “informal” (Kuffer, Pfeffer, & Sliuzas, 2016). We use “regular” and “irregular” because they are available in the SoAC data and because “formal” and “informal” definitions based on land ownership are not as applicable in our study areas which have mostly informal settlements (GoIRA & UN Habitat, 2015).

**Table 1:** Reclassification of land use subtypes to produce regular and irregular settlement types. Subtype categories come from the State of Afghan Cities report (GoIRA & UN Habitat, 2015) and are used to select locations to train the classification algorithm. Non-residential and uninhabited areas are not included in the classification.

Regular settlement	Irregular settlement	Non-residential/uninhabited
Regular	Irregular	Commercial
Apartments	Hillside houses	Industrial
Mixed use	IDP camps	Institutional
	Nomadic camps	Transport/roads
		Agriculture
		Under construction
		Vacant/barren
		Water/green space

The SoAC data are only available for municipal areas of each province, and, in some cases, do not cover the full extent of the built-up area outside of official municipal areas. In contrast, the settlement points dataset described below are complete for the whole of Afghanistan and the remote sensing data are likewise extensive, providing an opportunity to predict the settlement types beyond the SoAC study boundaries. To create training and validation data, the SoAC land use polygons are converted to match the 20 m processing grid. From within the reclassified land use rasters, 2000 locations (1000 each from regular and irregular housing areas) per province are randomly selected as training data. The land use rasters, excluding the training cell locations, are also used to validate the final prediction layers.

### 2.2 Settlement point data

Settlement points used in this study come from a dataset produced by Alcis Holdings Ltd. (www.alcis.org). Points locating residential compounds for all of Afghanistan were manually digitized from high resolution imagery between 2013 and 2014 by a team of specially trained, Afghan analysts. Imagery used for digitizing by the Alcis team was acquired primarily after January 2012. The country was divided into 1 km areas and assigned for digitizing work. Each area was digitized by two different analysts and the datasets compared and further reanalysed to ensure agreement. The seven provinces contain approximately 1.8 million settlement points (>40% of the entire country dataset). Points were projected to WGS84 UTM zone 42N prior to analyses. No additional attribute information, such as height, area, or building type are associated with the settlement points. Despite the limited attribute information of the settlement points, using a complete dataset that identifies residential compounds is an advantage over remote sensing analyses which must first identify settled or non-settled areas and then classify types of settlements while excluding non-residential areas.

### 2.3 Feature indices

Previous studies of settlement classification using VHR imagery or building polygons have identified a number of useful features to describe the orientation, spacing, density, and other physical characteristics of buildings in the urban landscape. The method presented here begins from the premise that a limited set of similar features extracted from the patterns of point locations of household compounds can provide sufficient information to distinguish settlement types. This work takes a multi-scalar approach to identifying settlement types by using a series of local moving windows of various sizes to calculate contextual features. First, a 20 m spatial resolution grid is created over the study area to define the extent of the classification. A buffer equal to the processing window radius is added to the province boundary when defining the study area to avoid edge effects. A circular filter is passed over the whole grid, iteratively centred on each grid cell. Settlement points located within the circular window at each location are selected for processing, and the results of the feature calculation are stored in the centre cell location before moving to the next output cell (see Supplementary material Section 1). The process is repeated for each radius of the search window which serves as the spatial scale of analysis. Radii included: 25, 50, 75, 100, 125, 150, 175, 200, 250, 300, 350, 400, 450, and 500 m.

We test nine different indices calculated from the point geometries (Table 2 Panel A). In addition to these geometry-derived features, we test an extension to the classification model that incorporates commonly available remote sensing data in addition to the geometry-derived features (Table 2, Panel B). These measures include elevation, slope, enhanced vegetation index (EVI), and nighttime lights from two different sources. These remote sensing data layers are publicly available and have been described in detail elsewhere for their use in population distribution models (Lloyd, Sorichetta, & Tatem, 2017). Below, the process for computing each geometry-derived feature is described before selecting the best performing measures in the variable selection and classification steps.

**Table 2:** Summary of all data layers considered in the study. Geometry-derived features (Panel A) are created from the Alcis Settlement Points dataset (www.alcis.org). Abbreviations are used when summarising the model results.

Data layers	Abbreviation
A. Geometry-derived features	
Number of points	npts
Unconstrained nearest neighbour mean distance	nd_m_f
Unconstrained nearest neighbour distance variance	nd_v_f
Constrained nearest neighbour mean distance	nd_m_c
Constrained nearest neighbour distance variance	nd_v_c
Linearity	l
Nearest neighbour index	ni
Nearest neighbour angle (Shannon's entropy)	na_s
Nearest neighbour angle (Metric entropy)	na_m

B. Remote sensing features	
Elevation	elev
Slope	slope
Enhanced vegetation index (EVI)	evi
Nighttime lights (VIIRS)	viirs
Nighttime lights (DMSP)	dmsp

#### 2.3.1 Number of points

The total number of settlement points found in the local search window. Within each level of the search radius, this measure becomes a point density. Certain other features can only be calculated with sufficient numbers of points in a local window, so this measure also acts as a logical branch to further processing.

#### 2.3.2 Nearest neighbour distance

The distance, in meters, for each settlement point to its first nearest spatial neighbour. The neighbours of each settlement are identified prior to the scanning window operations using a KD tree algorithm (Beygelzimer et al., 2013). The distance to the nearest neighbour is stored as an attribute to each settlement point, and the scanning window function calculates and stores the mean and the variance of the observed nearest neighbour distances as two separate features. Note that in the first form of this measure a point's nearest neighbour can be located anywhere in the study area. A separate data feature calculates the mean and variance of the distance between nearest neighbours only located within the circular processing window.

#### 2.3.3 Linearity

A summary of the dispersion of points within the local window and how much they tend to form a straight line. A rotationally invariant measure of linearity is calculated using the local covariance matrix of the two-dimensional point coordinates. Principal components analysis of this covariance matrix produces linearly uncorrelated, orthogonal eigenvectors, and the corresponding eigenvalues (*λ*), where *λ*_1_ ≥ *λ*_2_, contain information on the spread of the points in two directions. A measure of linearity, *L*, is calculated as:

(1) $$L=\frac{\lambda_{1}-\lambda_{2}}{\lambda_{1}}$$

Linearity values from Eq. (1) can range from 0, where there is equal spread in the two dimensions, to 1 where all points lie along a single line. Using local covariance matrices to derive linearity and other features has been used in processing 3D point clouds of scanned objects (Blomely, Weinmann, Leitloff, & Jutzi, 2014; Weinmann, Jutzi, Hinz, & Mallet, 2015), but, to our knowledge, has not been applied to describe geographic patterns before.

#### 2.3.4 Nearest neighbour index

The nearest neighbour index (NNI) compares the nearest neighbour distances, *d* for *n* points, observed within the local processing window of area, *A*, to a hypothetical mean distance expected if these points were randomly spaced in the same area (Eq. (2)).

(2) $$\mathrm{NNI}=\frac{\frac{\sum_{1}^{n}d_{i}}{n}}{.5\sqrt{\frac{A}{n}}}$$

The NNI measure is a well-known summary statistic in geographic point pattern analysis (Diggle, 2014) and was used by Mesev (2005) as a way to differentiate between urban neighbourhoods from point locations. The NNI ranges from 0 to 2.149, corresponding to spatially clustered and evenly dispersed, respectively. Values of NNI close to 1 indicate spatial randomness. The maximum of 2.149 occurs when points are uniformly spaced. Note that the NNI is highly sensitive to the choice of *A*, which in our processes is set by the spatial scanning window.

#### 2.3.5 Angle to nearest neighbour

This measure summarises the direction and patterns between neighbours in a local window. During the pre-processing step of calculating nearest neighbour distances between all points, the angle (expressed in degrees) to that neighbour is also stored as a settlement point attribute. The value of the angle, ranging from 0° to 359°, is collected into 45° intervals, which are further grouped into four diametrically opposed bins (i.e. 0°–45° is matched with 180°–225°) to identify straight line pairs of neighbours. For sensitivity testing the grouping process is repeated with the bins shifted by 22.5° to ensure differences are not due to the choice of bins. Two features are calculated to summarise the distribution of nearest neighbour angles in the moving window. The first is Shannon's entropy, *H(x)*, a measure of the diversity of angles.

(3) $$H\left(x\right)=-\sum_{i}\log_{2}\left(P\left(x\right)\right)$$

where *P(x)* is the observed proportion of nearest neighbour angles in each of the four bins. The second feature used is Metric entropy, which is *H(x)* scaled by the number of observations, in this case the number of settlement points in the local window. Entropy measures are more commonly used in information theory or ecology. In these measures, low values of entropy correspond with more orderly settlements as there is a lower diversity in the observed nearest neighbour angles. Graessar et al. (2012) used entropy measures of the length and orientation of line features extracted from imagery. While their measure characterises orientation and building layout, the distribution of angles between neighbouring settlement points characterises the degree of regularity in building locations.

### 2.4 Feature calculations

Each of the nine geometry-derived features described above is calculated for all search window sizes (14 different radii), and the combination of feature and spatial scales produces a total of 126 grids at 20 m spatial resolution covering the study area. The feature calculations are performed only in areas where settlement points are found. While the density of points and the unconstrained nearest neighbour distances and angles are calculated when at least 1 point is found in the processing window, the remaining features are only calculated when 3 or more points are found in the search window. Despite this restriction, given the number of settlement points in the study area and the fine resolution of the output grid, the process of calculating these features is computationally intensive. To improve efficiency of calculating the features, we divide the study area into sub-scenes, process each smaller grid in parallel, and remerge the grids of results before modelling. Computational load for these sub-scenes depends on 1) the number of settlement points and, 2) the number of pixels. Therefore the sub-scenes are split recursively to meet these two size criteria (see an example in the supplemental material section 1) and a buffer of cells from the adjacent sub-scenes equal to the search window radius must be added to avoid edge effects in the calculations. All calculations were performed in R 3.3.1 (R Core Team, 2016) using spatial data analysis (Bivand, Hauke, & Kossowski, 2013; Bivand, Keitt, & Rowlingson, 2017; Bivand & Piras, 2015; Hijmans, 2016) and visualisation packages (Wickham, 2009). Parallel processing steps for feature calculation, permutation tests, and spatial predictions used Rmpi (Yu, 2002) on OpenMPI version 2.0.0 (https://www.open-mpi.org/). An example script implementing the feature calculations is shown in the Supplementary material section 2.

### 2.5 Random forest classification and prediction

At each training location, the values for all layers are extracted, so each training site contains a multi-scale signature of the features. These attributes are used in a RF classifier to predict whether a location is of regular or irregular residential type. The RF, initially described by Breiman (2001), is an ensemble machine learning technique that uses the aggregated predictions from a large number of decision tree models. Each tree is grown independently using a sample of the data and at each decision node, covariates are randomly subset before the best split is selected. The result is a methodology which is flexible to find complex, non-linear relationships, yet robust to overfitting. Few parameters are needed to tune the RF: the number of trees to grow (*ntree*) and the number of covariates to select (*mtry*) at each branching step. In all tests we grow 1000 trees to ensure stable estimates. The number of covariates to select is set where $\mathrm{mtry}=\sqrt{\mathrm{number of features}}$, which is commonly used and found to perform well in many cases (Svetnik et al., 2003).

An important part of this study is to explore which features and which spatial scales are most useful for classifying settlement types and to find a parsimonious model. Most RF applications use a variable importance score to select variables from a large number of candidate predictors, either in a screening step or as part of a stepwise procedure. This score may be a mean decrease in accuracy calculated from the difference in prediction accuracy before and after permutating the predictor and normalized by the standard deviation across all trees. A second common importance score in classification models is the mean decrease in a Gini coefficient that describes how a variable contributes to splitting a decision tree into more homogenous nodes. More important variables will produce larger losses in accuracy and in node purity. However, a weakness of the typical RF approach is that when variables are highly correlated or when variables of different types or scales are mixed in a single model, the variable importance scores may be misleading. Strobl, Boulesteix, Zeileis, and Hothorn (2007) and Strobl, Boulesteix, Kneib, Augustin, and Zeileis (2008) show in several simulation studies that, among highly correlated variables, the random selections can lead to inconsistent decisions to include a variable. Moreover, when comparing different variables, those with more categories and continuous variables will appear to be preferred by typical variable importance scores. To account for these properties, random forests implementing subsampling without replacement as well as variable importance scores which condition on the variable type (Strobl et al., 2007, Strobl et al., 2008) and incorporate multiple permutations (Altmann, Toloşi, Sander, & Lengauer, 2010) have been suggested to avoid biases.

Our geometry-derived features, calculated across spatial scales do exhibit strong correlations. Additionally, by using the geometry-derived features and remote sensing layers, we are combining variables with different response scales. Therefore we implement a multi-step variable selection procedure using the unbiased, conditional random forests implemented in the cforest function and the permutation variable importance implemented in the varimp function, both of which are from the *party* package for R (Hothorn, Buehlmann, Dudoit, Molinaro, & Van Der Laan, 2006; Strobl et al., 2007, Strobl et al., 2008). In our procedure we first run a RF model 10 times starting with different random seeds while including all possible 126 geometry-derived features. We remove from further tests any variable whose minimum variable importance score for any repetition is zero. Second, we implement a version of the permutation test suggested by Altmann et al. (2010) and discussed by Hapfelmeier and Ulm (2013). An RF model is run using variables that remain after the first step to establish a baseline of variable importance scores measured as the mean decrease in accuracy. Then the settlement classification is randomly permutated 500 times and the model refit to each permutation while the predictor variables associated with the training observation remain unchanged. The variable importance scores are stored for each of these runs. If variables are related to the outcome, this permutation step should break that association and allow us to test if the observed variable importance scores from the baseline RF model occurred by chance. We approximate p-values for the variable importance score by comparing the observed score for the baseline model with the distribution of scores from the 500 permutation tests. Variables are considered “significant” at the p = .01 level. These steps establish a subset of the most important geometry-derived features that are retained for the final model. In the final selection step, we compare the out-of-bag (OOB) error rate for an RF model using this subset of features to one which also includes the remotely sensed features. The OOB is an internal measure of accuracy based on repeated random samples of the training data taken during model fitting. Comparing the prediction for non-selected training data at each iteration estimates an error rate. Lower OOB rates per prediction class suggest an improved model.

The resulting RF model is used to predict the discrete, two-type residential settlement class at the pixel level for each of the seven provinces containing training data. The prediction for the province was performed by applying the final, fitted RF model to the stack of feature layers, producing a 20 m gridded surface with regular and irregular settlement classes, followed by a 7 × 7 majority smoothing filter. Areas without settlement points (which includes both unsettled land and industrial/commercial areas) are not classified (shown in our final maps as whitespace). Accuracy of the prediction is assessed with the SoAC data as ground truth.

## 3 Results

### 3.1 Feature calculations

**Fig. 2:**
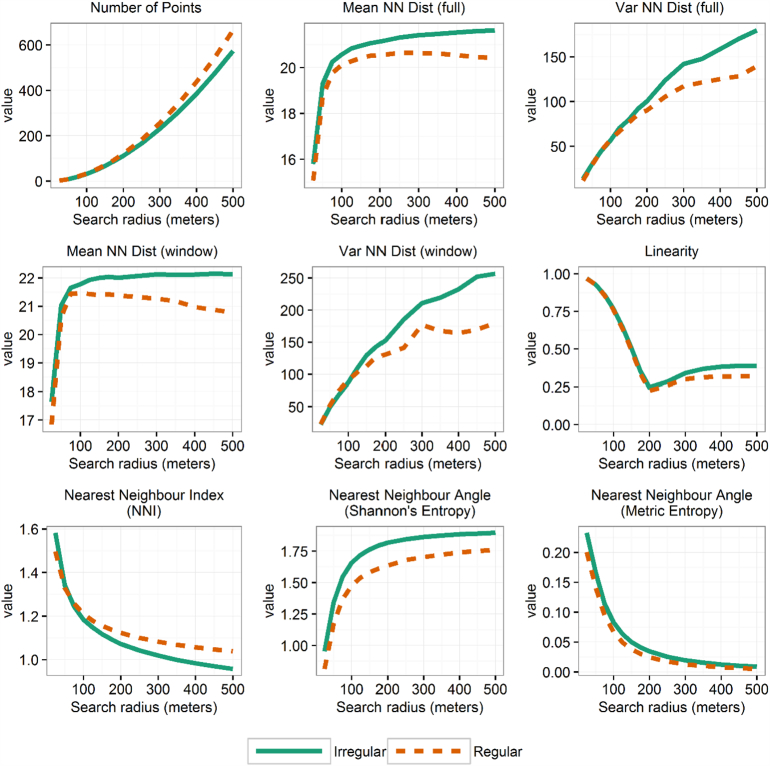
Geometry-derived features across spatial scales. Average values extracted from 14,000 training point locations for regular (dashed, gold coloured) and irregular (solid, green coloured) settlement areas. Residential types defined by the State of Afghan Cities (GoIRA & UN Habitat, 2015) land use parcels. (For interpretation of the references to colour in this figure legend, the reader is referred to the web version of this article.)

**Fig. 3:**
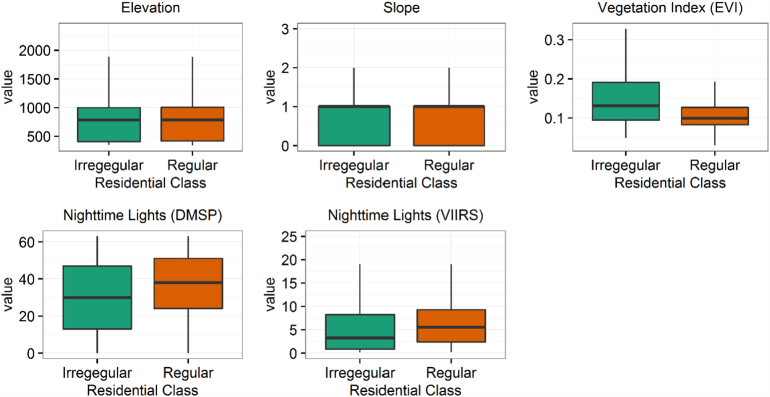
Remote sensing measures. Boxplots of values extracted at 14,000 training locations for regular (gold coloured) and irregular (green coloured) residential types. Residential types defined by the State of Afghan Cities (GoIRA & UN Habitat, 2015) land use parcels. (For interpretation of the references to colour in this figure legend, the reader is referred to the web version of this article.)

Feature calculations produced 131 data layers (126 geometry-derived layers and 5 remote sensing measures) for each province. The calculations required almost 57 h in total using 96 processors (on 6, 16-core computing nodes). Values from these layers were extracted at the randomly selected locations to create the training dataset. Fig. 2 shows the average signatures for features across spatial scales in regular and irregular residential types from the training data. Fig. 3 shows boxplots comparing the remote sensing measures between housing types at the training locations. Compared to regular types, irregular settlement areas tend to have slightly larger separation between neighbours, as seen in the mean distances. Irregular settlements also have higher entropy scores of nearest neighbour angle, suggesting, as expected, a less ordered arrangement of structures. Correspondingly, regular settlements show higher values of the NNI, suggesting more evenly dispersed settlement points. Based on the remote sensing data, both settlement types tend to be found at lower elevations and relatively flatter areas. Irregular settlements are in greener areas based on EVI while regular settlements were found to have higher values of (i.e. brighter) nighttime lights in both VIIRS and DMSP.

### 3.2 Variable selection

**Fig. 4:**
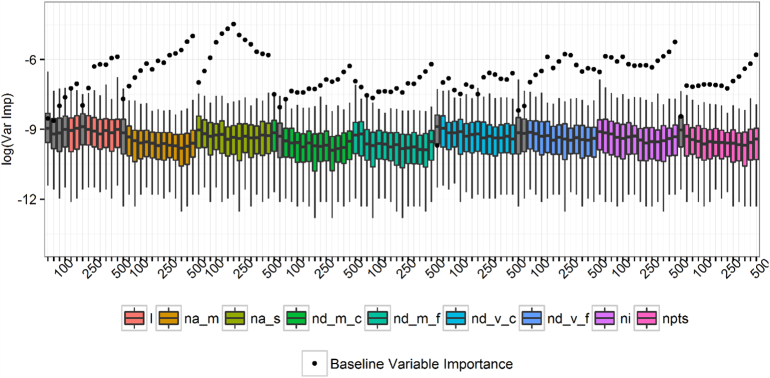
Variable importance and feature selection among geometry-derived features. The variable importance score observed in the baseline random forest model is compared to results from 500 random permutations. Non-significant variables at p = .01 level are shown in grey. p-Values are approximated by comparing the baseline variable importance to the distribution of permutations. Feature abbreviations are given in Table 2. (For interpretation of the references to colour in this figure legend, the reader is referred to the web version of this article.)

The first processing step based on 10 runs of the RF suggested dropping the linearity and both nearest neighbour angle measures at the 25 m scale. In the second step, we entered the remaining 123 geometry-derived features in a RF model and compared the resulting baseline variable importance to those from 500 random permutations. The results of this test are shown in Fig. 4. The distribution of variable importance from the permutations is shown as a boxplot and compared with the observed, baseline point observation. Eleven features (scales): l (50, 75, 100, 125, 200), na_m (50), nd_m_c (50), nd_v_c (25), nd_v_f (25, 50), npts (25) are discarded based on p-values exceeding the 0.01 threshold (Fig. 4). Using this subset of geometry-derived features in a RF model produced an overall 84.9% OOB accuracy rate. For comparison a RF model using all 126 geometry-derived features (i.e. ignoring all variable selection steps) achieved an 83.8% OOB accuracy rate. The feature signature plots and the first two variable selection steps consistently identify the most important variables for classifying the settlement types to be from processing scales above 100 m. In general, the entropy measures of nearest neighbour angles and the nearest neighbour index ranked among the most important variables. Results of additional sensitivity tests (not shown) comparing the OOB error rates after removing all features calculated at 100 m or less, also support this idea. Note also that these results were not sensitive to rotating the bins when defining the nearest neighbour angle. By incorporating the remote sensing measures along with the final subset of geometry-derived features, the RF achieved an 89.6% overall OOB accuracy rate. We also tested adding the remote sensing variables one at a time and in different combinations with the selected geometry-derived features. The OOB accuracies for these models ranged from 83.8% to 85.7%. Therefore the final model used for prediction excluded geometry-derived features calculated at 100 m or less, linear features at 125 and 200 m, and included all the remote sensing features.

### 3.3 Classification and accuracy assessment

**Fig. 5:**
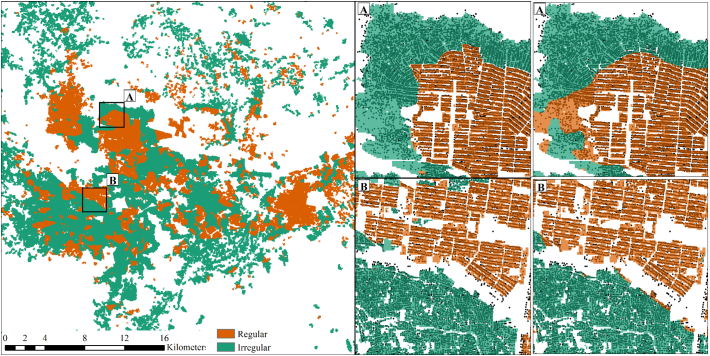
Example classification results for the city of Kabul using a 7 × 7 smoothing function on the prediction results. Insets show two areas of the city with validation data and settlement points used to calculate the geometry-derived features (Left column) and the prediction map of regular and irregular residential types (Right column). Whitespace in the maps are unsettled or non-residential areas. (For interpretation of the references to colour in this figure legend, the reader is referred to the web version of this article.)

We report the cross-tabulation of pixel-level predicted vs. SoAC residential types as well as positive and negative predictive values, sensitivity, specificity and overall accuracy measures for the predictions in each province. A summary of the main results is given in Table 3. Panel A of the table gives the prevalence of each type in the validation data (based on pixel counts). The prevalence of regular settlement area ranged widely in the validation datasets from 14% in Kunduz to 60% in Balkh where the provincial capital is Mazar-i-Sharif. Panel B reports the cross-tabulation counts of a pixel-by-pixel comparison between the prediction and validation data. For convention, we use the language of “positive” and “negative” for our binary classifications, with regular settlements referred to as positive. Therefore a true positive is a correctly predicted regular settlement pixel and a true negative is a correctly predicted irregular settlement pixel. Panel C reports the accuracy assessments from the cross-tabulations. Positive predictive value (PPV) and negative predictive value (NPV) refer to percent of correctly predicted regular and irregular settlement pixels. Note that PPV and NPV depend on the prevalence of each settlement type and the results follow these patterns. Sensitivity and specificity are not affected by the prevalence, and the model shows good ability to correctly predict the settlement type across the provinces. Sensitivity, a measure of correctly classified regular settlement pixels, ranges from 71.1% in Herat to 91.9% in Helmand. Specificity, a measure of correctly classified irregular settlement pixels, shows slightly better performance ranging from 79.4% in Kandahar to 93.4% in Nangarhar. Overall accuracy (calculated as the sum of true positives and true negatives divided by the total number of pixels) provides a summary measure and ranged from 77.6% in Kandahar to 90.3% in Nangarhar. An example of the mapped classification results are shown in Fig. 5 for the city of Kabul. The final classification map includes a majority smoothing after prediction. The inset images of the figure show an example of the gridded, reclassified SoAC data used for training and validation overlaid on the settlement points used for feature calculations. The right most panels of Fig. 5 show an example of the predictions only in the validation locations as used to evaluate the classifier. Whitespace is non-settled or non-residential areas. Final prediction maps for the other provincial capitals are included in the Supplementary material (Section 3).

**Table 3:** Classification accuracy. The confusion matrix compares residential types not used for training with the predicted classification from the final random forest model (which include geometry-derived and remote sensing predictors) at a pixel level. Validation data come from the reclassified State of Afghan Cities report (GoIRA & UN Habitat, 2015) from the provincial capitals in each of the seven provinces.

	A. Settlement type prevalence		B. Predicting regular settlement (pixels)				C. Assessment				
	Regular	Irregular	True positive	False positive	True negative	False negative	PPV^a^	NPV^b^	Sensitivity^c^	Specificity^d^	Overall accuracy
Balkh	60.1%	39.9%	38,849	3767	24,356	3600	91.2%	87.1%	91.5%	86.6%	89.6%
Helmand	33.5%	66.5%	18,345	7007	32,673	1618	72.4%	95.3%	91.9%	82.3%	85.5%
Herat	38.7%	61.3%	22,706	7643	43,033	9225	74.8%	82.3%	71.1%	84.9%	79.6%
Kabul	28.3%	71.7%	87,581	43,287	263,375	33,553	66.9%	88.7%	72.3%	85.9%	82.0%
Kandahar	45.7%	54.3%	31,810	10,322	39,812	10,304	75.5%	79.4%	75.5%	79.4%	77.6%
Kunduz	14.1%	85.9%	4170	2795	27,053	722	59.9%	97.4%	85.2%	90.6%	89.9%
Nangarhar	38.6%	61.4%	17,862	2211	31,062	3027	89.0%	91.1%	85.5%	93.4%	90.3%

a PPV (positive predictive value) = true positive / (true positive + false positive).

b NPV (negative predictive value) = true negative / (true positive + false negative).

c Sensitivity = true positive / (true positive + false negative).

d Specificity = true negative / (true negative + false positive).

## 4 Discussion and conclusions

Creating consistent and objective land use maps for large, growing urban areas remains a challenge, but these maps are needed for monitoring growth and urban planning and for development projects. Urban land use classifications can serve broader needs as well, such as inputs into estimating population density and distribution (Linard, Gilbert, Snow, Noor, & Tatem, 2012; Tatem & Hay, 2004). While VHR imagery is most commonly used to monitor urban areas, vector data are often now collected in sufficient detail to present an alternative approach to measure the physical structure of cities and differentiate land uses. With few exceptions, previous work on using urban morphology to determine land use has used 2D and 3D representations of buildings. This work has demonstrated the potential of measuring characteristic neighbourhood features in simple point representations and to use those features when classifying areas into residential settlement types. We do not suggest that this approach should replace imagery analyses or other, richer vector data, but that creative analyses can effectively make use of existing resources.

We tested nine different features calculated from the point geometries (number of points, mean and variance of nearest neighbour distance, linearity, NNI, entropy of nearest neighbour angles) across 14 spatial scales. In order to find the most important variables we implemented a multi-step selection process. The trade-off for these decisions was increased computational time. We were able to accomplish these calculations by splitting the data and processing them in parallel in a high performance computing environment, but this approach would not be feasible with only desktop computing resources. The conditional random forest method is substantially slower than the more commonly used random forest functions (Liaw & Wiener, 2002), and the permutation tests to estimate p-values took several hours running in parallel on a 16-core computer. From the results, the nearest neighbour angles and the NNI were consistently among the most important variables as measured by mean decrease in accuracy in group analyses and spatial scales above 100 m for all measures tended to also be relatively more important. Mesev (2005) also found good results using the NNI in his study of address points in the UK, but he did not examine different spatial scales. The subset of geometry-derived features provided good accuracy in OOB tests which was further improved when remote sensing measures of elevation, slope, vegetation, and nighttime lights were included. The more parsimonious RF model outperformed a model including all possible covariates. Overall accuracies of the final RF model were high and ranged between 78% and 90% in seven provincial capitals of Afghanistan. At the suggestion of two reviewers we also implemented a maximum likelihood (ML) classifier using the same set of training data for comparison. We tested the ML method in Kabul and Kandahar and achieved accuracies of only 63.9% and 67.2%, respectively. While the ML method is simpler to implement, the RF method is able to find more complex and non-linear relationships which likely improves its performance.

From visual inspection of our results, most classification errors occur at the boundaries between settlement types, and the model performs best in areas with large, contiguous areas of each type. Therefore the overall form of the urban areas will impact the performance of the model and may partly explain the variation in accuracy among our seven study areas. Province-specific models may improve performance, but we sought a more general solution. Graessar et al. (2012) produced a formal vs. informal neighbourhood classification for Kabul from VHR imagery. Our patterns of the classified residential areas largely align visually with their published map, with large areas of regular settlements in the north and east of the city, though we note we are working with different land use definitions and exclude non-residential areas from our maps. Afghanistan has experienced very rapid urbanisation over the past 15 years. The growth of cities with limited oversight has produced many vacant plots and fragmented development (French, Turkstra, & Farid, 2016). Sparse settlements and greater distances between settlement points make it more difficult to determine regular settlement types in particular using our method. While the time period for our settlement points and training/validation data generally align, rapid changes could cause some differences in the land use classification and observed points, particularly around new developments on the urban fringe. Because the settlement point dataset we used was manually digitized, there are potentially errors of omission and commission of residential buildings, as well as inconsistencies in the point positions atop each structure. We made no adjustments in the settlement point data, and these differences did not prevent the classification from reaching good levels of accuracy.

Our definitions of regular and irregular settlements for training and validation were based on the SoAC dataset (GoIRA & UN Habitat, 2015), which is unrelated to the creation of the settlement points. This external validation is an advantage of the study, though the classification of residential types was limited to these two types. We did not use the terminology of informal (or slums) as other urban land use classification studies have used. Informal settlements refer to land ownership, and in our study area the vast majority of urban settlements are informal as a result of land grabbing and unlicensed development even though they may appear somewhat formal by their settlement patterns (GoIRA & UN Habitat, 2015). Irregular settlements which are defined by spatial patterns may be a more relevant term in this setting to identify areas to prioritise for urban planning and development. Additionally, these SoAC classes are based on land uses in the provincial capitals. The meaning of any settlement type in rural areas and smaller cities is less clear. Our classification maps identify regular and irregular areas based on the point patterns, but other, in-depth studies would be needed to understand their function within rural areas or other contexts. An alternative analytical strategy could explore unsupervised classification of the point pattern features to see if a more nuanced settlement typology can be extracted.

This work has focused on the geometry features that can be extracted solely from point patterns, and we have only tested a limited number of simple metrics from among many potential. In particular, the scanning window scale and constraint should be explored further, such as with a window-constrained nearest neighbour angle measure. Another possible enhancement to the method is to look at the settlement points in relation to other features of the built and natural landscapes. The relative positioning to major roads, intersections, or water features all could contribute to improved classifications. Future work should also explore neighbourhood classification by fusing the two approaches of feature information derived from vector data with those features that can be extracted from high resolution imagery.

More broadly, this work provides an example of extracting information from big data to support geographic research. Such data sources require researchers to not only utilise new computational methods to make use of such resources but to have creative and alternative interpretations of the data. Large collections of spatial point locations may become more common as censuses and other routine data collections are geolocated to household locations. Thus, there may be more opportunities in the future to use point pattern analyses in combination with other data sources to improve our understanding of populations.

## Appendix A Supplementary material

Supplementary material and example code. Image 1

Sample data. Image 2
