# Supplementary material for: Identifying residential neighbourhood types from settlement points in a machine learning approach
Source: Comput Environ Urban Syst. 2018 May;69:104–13. doi: 10.1016/j.compenvurbsys.2018.01.004 (PMC5863080; doi:10.1016/j.compenvurbsys.2018.01.004)

**Supplementary Material for *Identifying residential neighbourhood types from settlement points in a machine learning approach***

**Section 1: Processing and Computational Methods**

*Overview of processing steps*

The geometry-derived features are calculated using a moving window operation. A circular filter with a fixed radius moves across each cell of the 20 m resolution output grid. At each step, settlement points within the circular window are selected and used to calculate various features as described in the text. Each feature is stored in a separate gridded layer. The entire process is repeated for each radii.


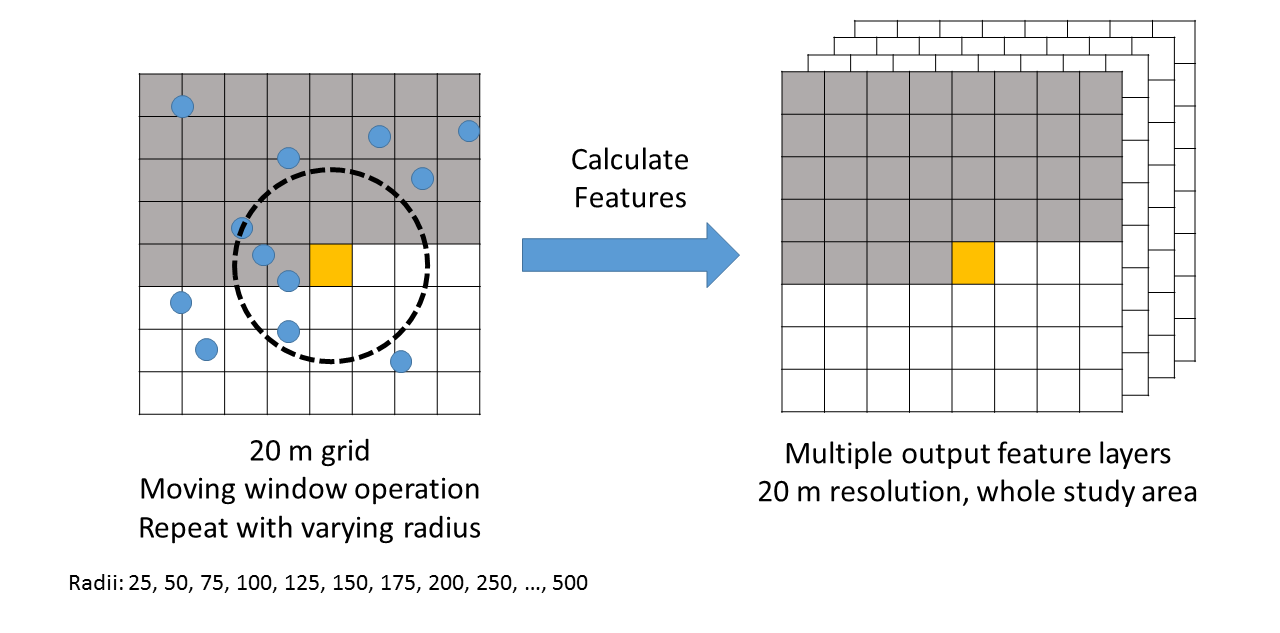


Figure: Example processing steps to calculate geometry-derived features.

*Sub-scenes within the study area*

The process described above is computationally intensive given the number of radii and the large area being analysed. To improve computational efficiency of feature calculations, a recursive splitting algorithm is used to divide the study area into smaller, sub-scene areas that balances the size of the scene with the number of settlement points in the area. The smallest blocks correspond to areas with higher densities of settlement points. An example of these splits is shown in Figure 7. The smallest blocks correspond to urban areas of the provincial capital with a large number of settlement points. By adding a “halo” of cells around each sub-scene equal to the size of the processing radius, each sub-scene can be processed in parallel while avoiding edge effects in the moving window calculation.


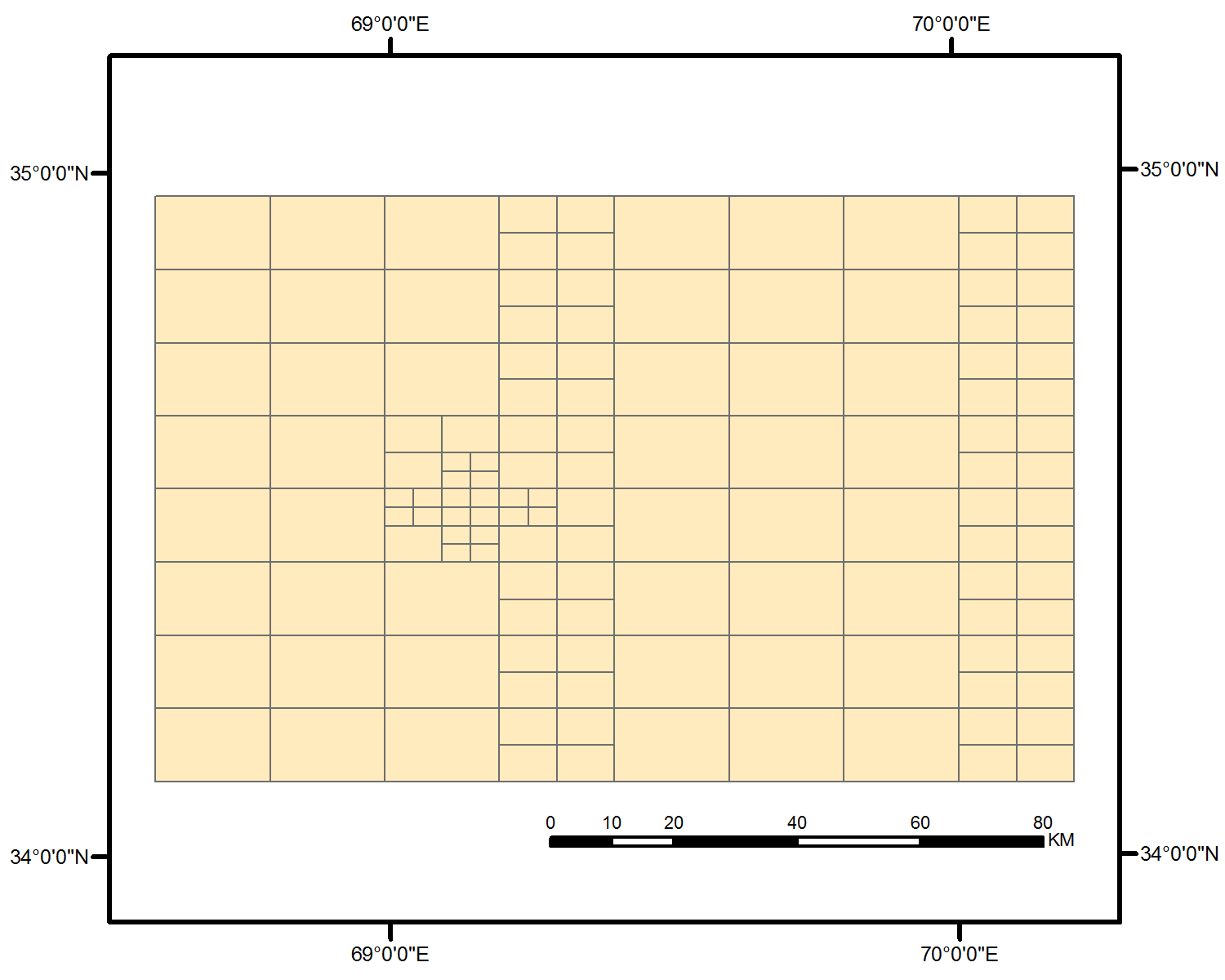


Figure 7: Example sub-scene splitting covering the province of Kabul.

**Section 2: R Code demonstration of point pattern feature calculations**

#

# Example feature calculations from settlement points

#

# This code demonstrates the general ideas of calculating

# data features from spatial point patterns used in the

# paper "Identifying residential types from settlement

# points in a machine learning approach."

#

# Calculations use only 4 spatial scales for demonstration.

# Settlement points are not actual locations.

#

require**(**spdep**)**

require**(**FNN**)**

require**(**raster**)**

# read settlement point locations

# change to your file path

setwd**(**"C:/Users/CHANGE/PATH/HERE/"**)**

pts **<-** read.csv**(**"settlement_pts_example.csv",

stringsAsFactors**=**F**)**

str**(**pts**)** # uid=unique id, x/y=locations

dim**(**pts**)** # n=9004

head**(**pts**)**

# plot(pts$x, pts$y)

# pre-calculate nearest neighbour distances and angles

# used for unconstrained feature calculations

kn1 **<-** get.knn**(**pts**[**,c**(**"x","y"**)]**, k**=**1**)**

pts**$**dist **<-** kn1**$**nn.dist

# use neighbour pairs to find angles

ptpair **<-** data.frame**(**pts**[**,c**(**"x","y"**)]**, pts**[**kn1**$**nn.index, c**(**"x","y"**)])**

names**(**ptpair**)** **<-** c**(**"x1","y1","x2","y2"**)**

# make angles

ptpair**$**dx **<-** ptpair**$**x2 **-** ptpair**$**x1

ptpair**$**dy **<-** ptpair**$**y2 **-** ptpair**$**y1

ptpair**$**a **<-** atan2**(**ptpair**$**dy, ptpair**$**dx**)** ***** 180**/**pi

ptpair**$**a **<-** ptpair**$**a %% 360

# bin the angles into 4 groups

ptpair**$**b **<-** as.numeric**(**cut**(**ptpair**$**a,

breaks**=**c**(**0,45,90,135,180,225,270,315,360**)**,

include.lowest**=**T**))**

ptpair**[**ptpair**$**b**==**1 **|** ptpair**$**b**==**5, "b"**]** **<-** 1

ptpair**[**ptpair**$**b**==**2 **|** ptpair**$**b**==**6, "b"**]** **<-** 2

ptpair**[**ptpair**$**b**==**3 **|** ptpair**$**b**==**7, "b"**]** **<-** 3

ptpair**[**ptpair**$**b**==**4 **|** ptpair**$**b**==**8, "b"**]** **<-** 4

# merge back to the original

pts **<-** cbind**(**pts, ptpair**[**,c**(**"a","b"**)])**

# create a high-resolution grid on which to calculate features

# dimensions are set based on the example settlement point data

grid **<-** raster**(**nrows**=**130, ncols**=**130, xmn**=**0, xmx**=**2600, ymn**=**0, ymx**=**2600**)**

# coordinates of each cell

gridpts **<-** coordinates**(**grid**)**

# spatial scales for analysis

spscales**<-**c**(**25,50,75,100**)**

####

# Calculate point pattern features

# loop each scale

**for(**r **in** spscales**){**

print**(**r**)** # radius of search

# pre-allocate output

output **<-** vector**(**mode**=**"list", length**=**nrow**(**gridpts**))**

# main processing loop -- over all grid cells

**for(**j **in** 1**:**nrow**(**gridpts**)){**

# calculate distances to subset by radius

dists **<-** spDistsN1**(**as.matrix**(**pts**[**,c**(**"x","y"**)])**,

matrix**(**gridpts**[**j,1**:**2**]**, ncol**=**2**)**,

longlat**=**F**)**

# select points within window for processing

subpts **<-** pts**[**which**(**dists**<=**r**)**,**]**

**if(**nrow**(**subpts**)==**0**){** # if empty, skip

output**[[**j**]]** **<-** data.frame**(**j**=**j,

npts**=-**1,

nnd_m_f**=-**1,

nnd_v_f**=-**1,

nnd_m_c**=-**1,

nnd_v_c**=-**1,

l**=-**1,

nni**=-**1,

nna_s**=-**1,

nna_m**=-**1**)**

**}** **else{**

**if(**nrow**(**subpts**)<**3**){** # limited stats

npts **<-** nrow**(**subpts**)**

nnd_m_f**=-**1

nnd_v_f**=-**1

nnd_m_c**=-**1

nnd_v_c**=-**1

l**=-**1

nni**=-**1

nna_s**=-**1

nna_m**=-**1

**}** **else{** # feature calculations

# number of points

npts **<-** nrow**(**subpts**)**

# nearest neighbour distances (unconstrained)

nnd_m_f **<-** mean**(**subpts**$**dist**)**

nnd_v_f **<-** var**(**subpts**$**dist**)**

# nearest neighbour distances (window-constrained)

kn1 **<-** knearneigh**(**as.matrix**(**subpts**[**,c**(**"x","y"**)])**,

k**=**1,

longlat**=**F**)**

dkn1 **<-** nbdists**(**knn2nb**(**kn1**)**,

coords**=**as.matrix**(**subpts**[**,c**(**"x","y"**)])**,

longlat**=**F**)**

avgnnd **<-** mean**(**unlist**(**dkn1**))**

nnd_m_c **<-** avgnnd

nnd_v_c **<-** var**(**unlist**(**dkn1**))**

# linearity

cv **<-** cov**(**subpts**)**

e **<-** eigen**(**cv**)$**values

l **<-** **(**e**[**1**]-**e**[**2**])/**e**[**1**]**

# nearest neighbour index

nni **<-** avgnnd **/** **(**0.5*****sqrt**((**pi*****r**^**2**)/**nrow**(**subpts**)))**

# nearest neighbour angles

p **<-** prop.table**(**table**(**subpts**$**b**))**

nna_s **<-** **-**sum**(**p*****log2**(**p**))** # shannon's entropy

nna_m **<-** **-**sum**(**p*****log2**(**p**))** **/** nrow**(**subpts**)** # metric entropy

**}**

output**[[**j**]]** **<-** data.frame**(**j**=**j, npts, nnd_m_f, nnd_v_f,

nnd_m_c, nnd_v_c, l, nni, nna_s,nna_m**)**

**}**

**}** # end loop over grid cells

print**(**" creating rasters"**)**

# combine calculations

o **<-** do.call**(**rbind.data.frame, output**)**

# create grids

npts_grid **<-** rasterize**(**gridpts, grid, field**=**o**$**npts**)**

avgnnd_m_f_grid **<-** rasterize**(**gridpts, grid, field**=**o**$**nnd_m_f**)**

avgnnd_v_f_grid **<-** rasterize**(**gridpts, grid, field**=**o**$**nnd_v_f**)**

avgnnd_m_c_grid **<-** rasterize**(**gridpts, grid, field**=**o**$**nnd_m_c**)**

avgnnd_v_c_grid **<-** rasterize**(**gridpts, grid, field**=**o**$**nnd_v_c**)**

l_grid **<-** rasterize**(**gridpts, grid, field**=**o**$**l**)**

nni_grid **<-** rasterize**(**gridpts, grid, field**=**o**$**nni**)**

nna_s_grid **<-** rasterize**(**gridpts, grid, field**=**o**$**nna_s**)**

nna_m_grid **<-** rasterize**(**gridpts, grid, field**=**o**$**nna_m**)**

# write out for each feature

writeRaster**(**npts_grid, paste0**(**"npts_", r, ".tif"**)**, format**=**"GTiff",

NAflag**=-**1, datatype**=**'FLT4S', options**=**c**(**"COMPRESS=LZW"**))**

writeRaster**(**avgnnd_m_f_grid, paste0**(**"nnd_m_f_", r, ".tif"**)**,

format**=**"GTiff", NAflag**=-**1, datatype**=**'FLT4S',

options**=**c**(**"COMPRESS=LZW"**))**

writeRaster**(**avgnnd_v_f_grid, paste0**(**"nnd_v_f_", r, ".tif"**)**,

format**=**"GTiff", NAflag**=-**1, datatype**=**'FLT4S',

options**=**c**(**"COMPRESS=LZW"**))**

writeRaster**(**avgnnd_m_c_grid, paste0**(**"nnd_m_c_", r, ".tif"**)**,

format**=**"GTiff", NAflag**=-**1, datatype**=**'FLT4S',

options**=**c**(**"COMPRESS=LZW"**))**

writeRaster**(**avgnnd_v_c_grid, paste0**(**"nnd_v_c_", r, ".tif"**)**,

format**=**"GTiff",

NAflag**=-**1, datatype**=**'FLT4S', options**=**c**(**"COMPRESS=LZW"**))**

writeRaster**(**l_grid, paste0**(**"l_", r, ".tif"**)**, format**=**"GTiff",

NAflag**=-**1, datatype**=**'FLT4S', options**=**c**(**"COMPRESS=LZW"**))**

writeRaster**(**nni_grid, paste0**(**"nni_", r, ".tif"**)**, format**=**"GTiff",

NAflag**=-**1, datatype**=**'FLT4S', options**=**c**(**"COMPRESS=LZW"**))**

writeRaster**(**nna_s_grid, paste0**(**"nna_s_", r, ".tif"**)**, format**=**"GTiff",

NAflag**=-**1, datatype**=**'FLT4S', options**=**c**(**"COMPRESS=LZW"**))**

writeRaster**(**nna_m_grid, paste0**(**"nna_m_", r, ".tif"**)**, format**=**"GTiff",

NAflag**=-**1, datatype**=**'FLT4S', options**=**c**(**"COMPRESS=LZW"**))**

**}** # end loop over scale

print**(**"Finished!"**)**

**Section 3: Prediction maps for seven provincial capital areas**


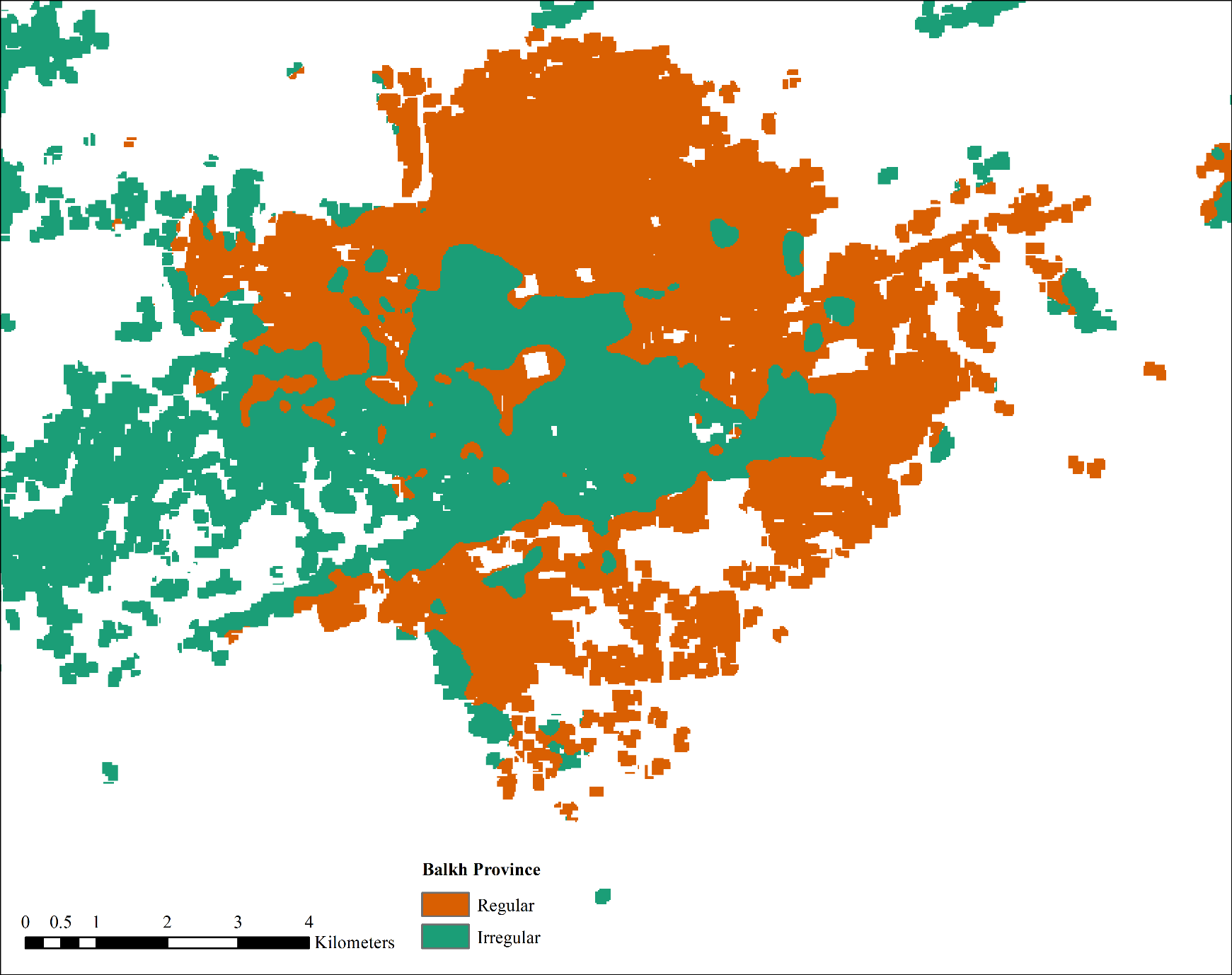

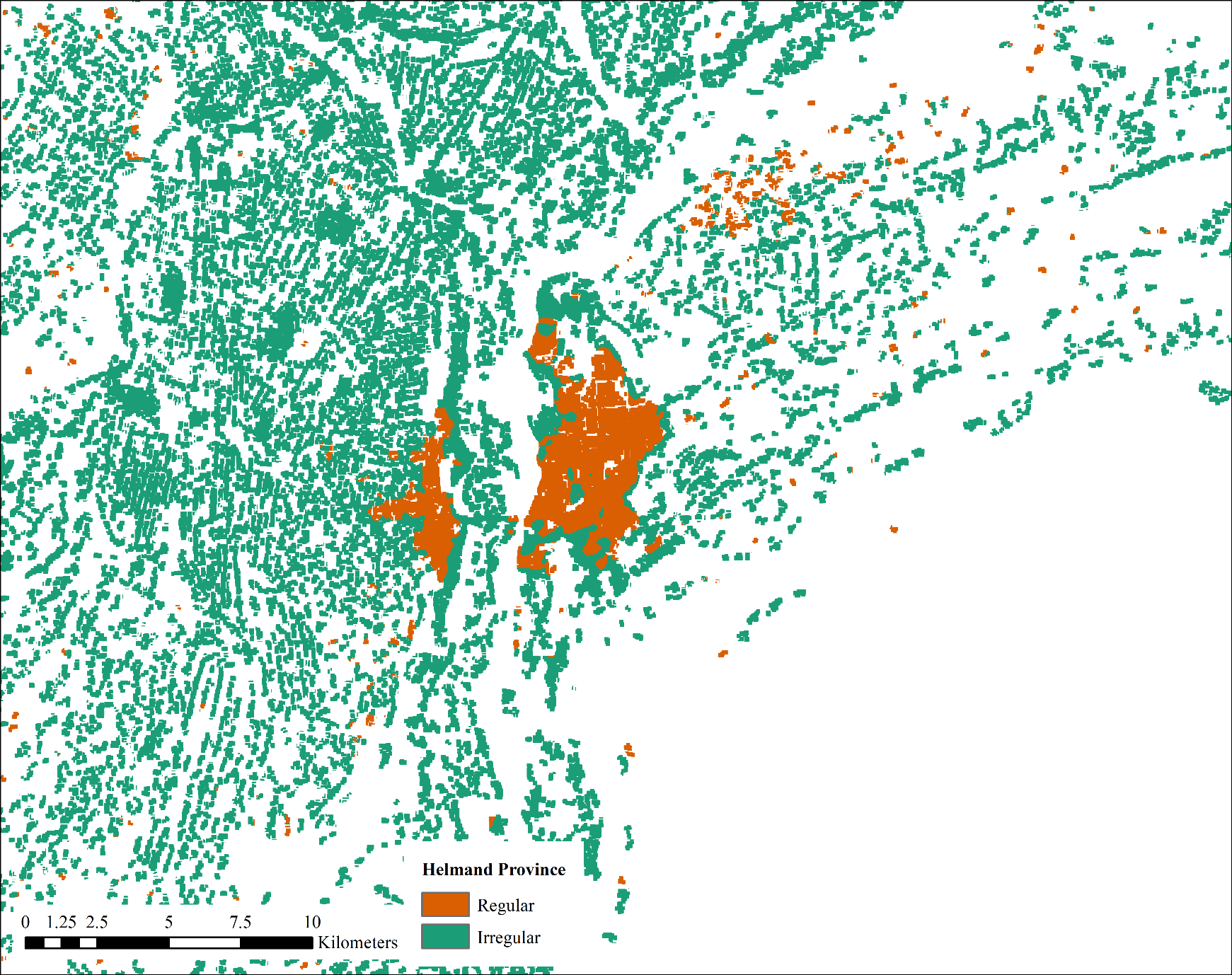

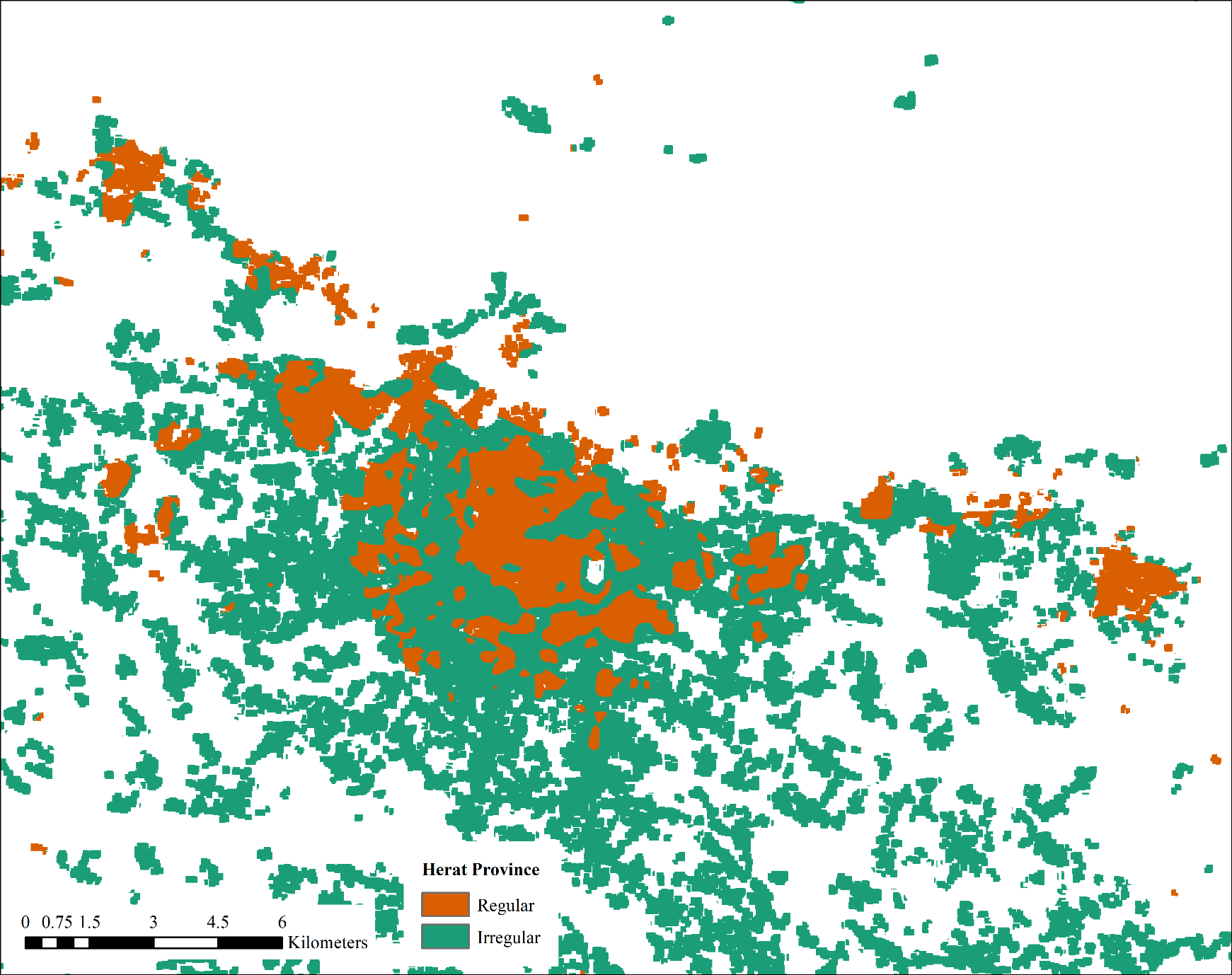

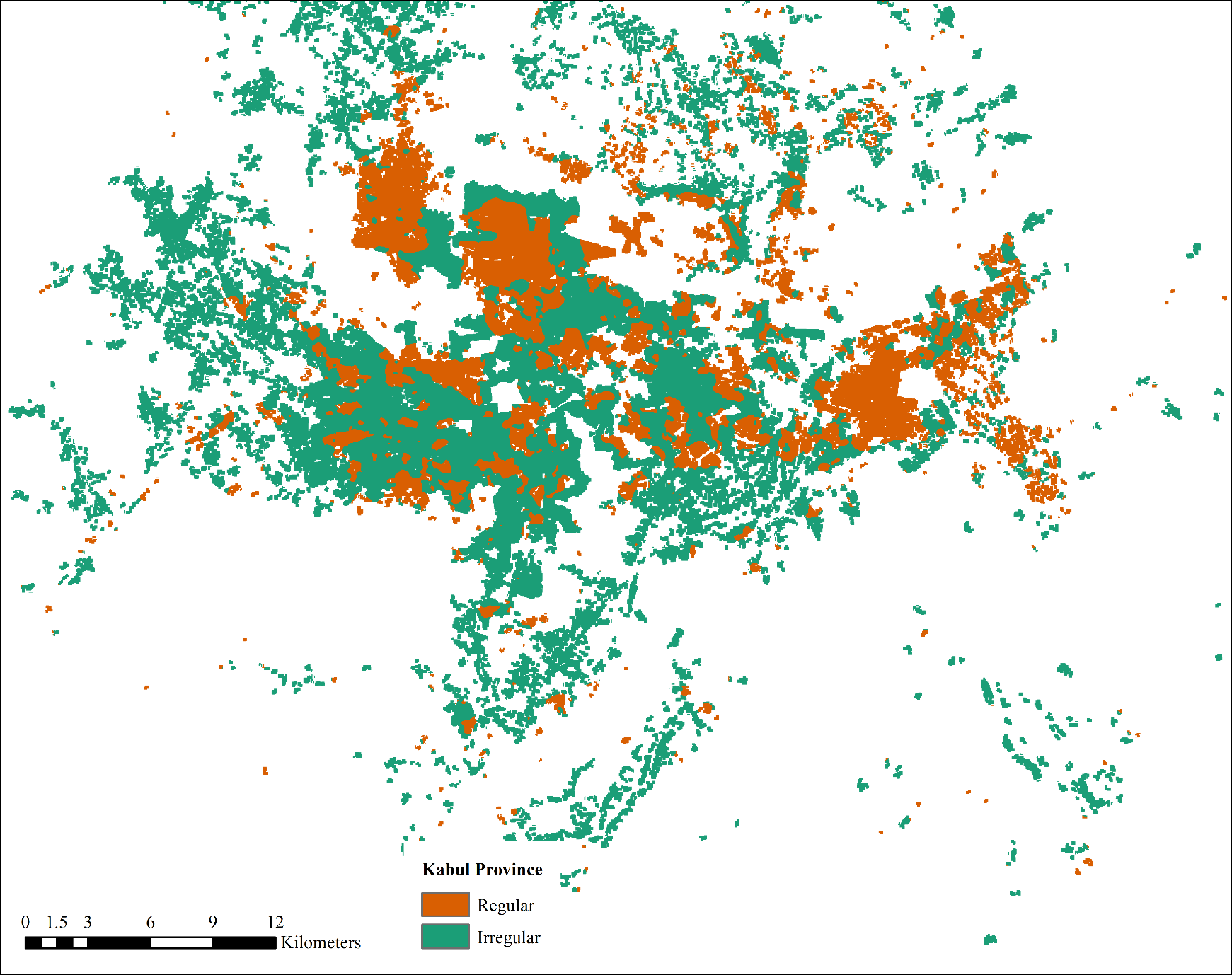

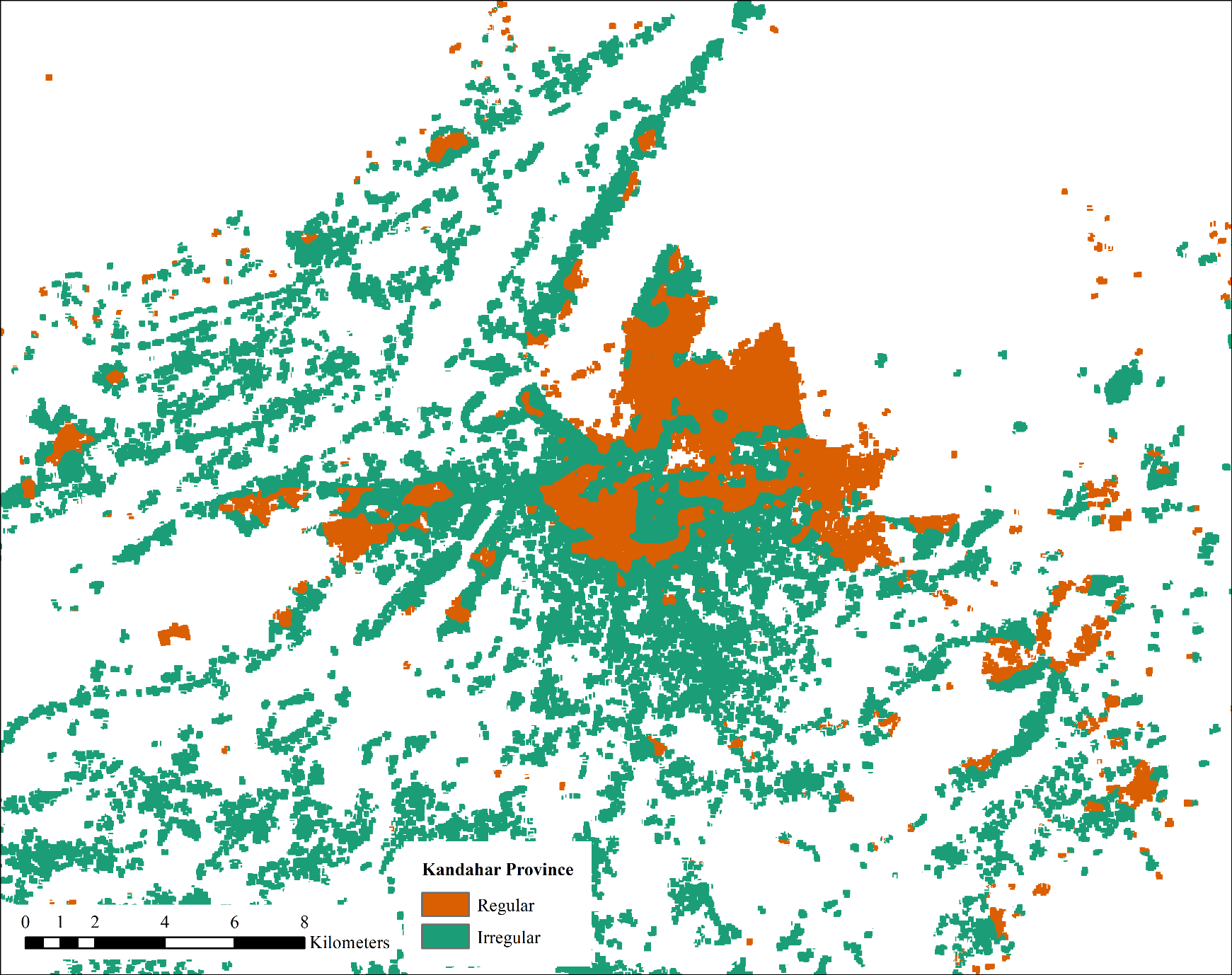

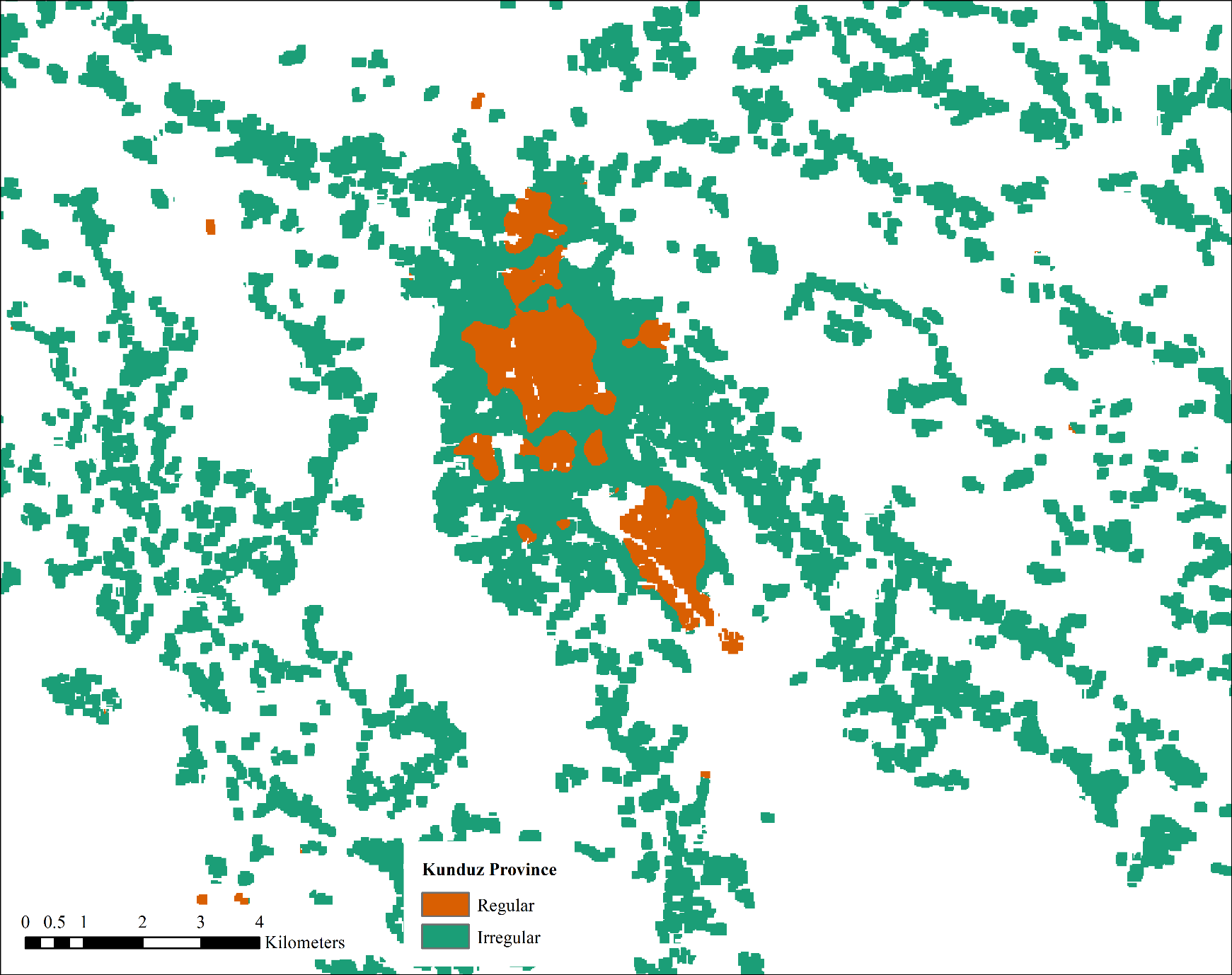

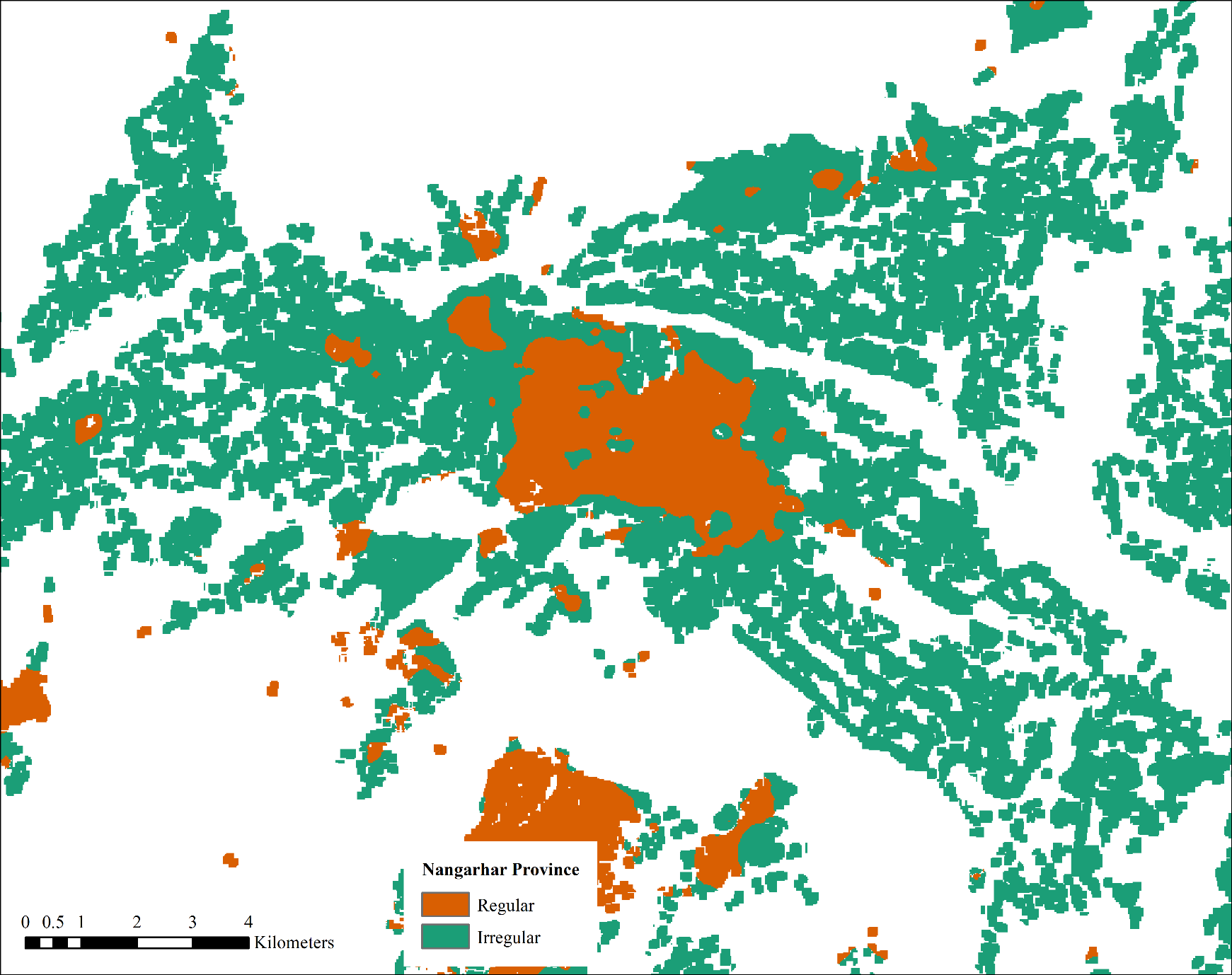

Supplement: Supplementary file 1 — Supplementary material and example code. [file mmc1.docx]
